# Expression of carbonic anhydrase IX suggests poor response to therapy in rectal cancer

**DOI:** 10.1038/sj.bjc.6605090

**Published:** 2009-06-30

**Authors:** P N Span, I D Nagtegaal, J Bussink

**Affiliations:** 1 Department of Radiation Oncology, Radboud University Nijmegen Medical Centre, Nijmegen, The Netherlands; 2 Department of Chemical Endocrinology, Radboud University Nijmegen Medical Centre, Nijmegen, The Netherlands; 3 Department of Pathology, Radboud University Nijmegen Medical Centre, Nijmegen, The Netherlands

**Sir,**

We have read with great interest the article by Korkeila *et al* (2009) ‘Expression of carbonic anhydrase IX suggests poor outcome in rectal cancer’ in a recent issue of the *British Journal of Cancer*. As the authors rightly state, CA-IX has been found to be of clinical value in different tumour types. The authors, however, state that their study was ‘designed to assess the *prognostic and predictive* value of CA-IX in rectal cancer treated by either short or long course of radiotherapy’. Although this might be the case, their data analysis does not support this statement.

The prognostic value of CA-IX, if present, cannot be deduced from the applied patient cohort, as all patients received radio- and/or chemotherapy. A pure prognostic factor can only be detected in an untreated patient population. CA-IX is also known to be strongly induced by hypoxia through HIF1a upregulation (Lal *et al*, 2001). The hypoxic phenotype that is represented by the high expression levels of CA-IX is very likely to cause a predictive value of CA-IX, predicting in this case resistance to radiotherapy and possibly also chemotherapy. Earlier, we have found CA-IX to be predictive for different adjuvant treatments in breast cancer (Span *et al*, 2003).

On the other hand, the authors also describe a control group that would seem to have received no neoadjuvant chemo- or radiotherapy. If these patients also did not receive adjuvant therapy, which is regrettably unclear from the paper, the authors should be able to determine whether the clinical value of CA-IX is similar in treated and untreated rectal cancer patients. This could add valuable information to this subject and would warrant publication.

In our opinion, the results shown so far only allow the statement ‘Expression of Carbonic Anhydrase IX suggests poor response to therapy in rectal cancer’.
